# Antimicrobial Resistance Profile of *Klebsiella pneumoniae* Clinical Isolates at a Tertiary Pulmonology-Critical Care Centre in Romania: A Five-Year Retrospective Study (2021–2026)

**DOI:** 10.3390/antibiotics15080774

**Published:** 2026-08-12

**Authors:** Loredana Sabina Cornelia Manolescu, Madalina Solomon, Oana Popescu, Beatrice Mahler, Dragos-Cosmin Zaharia

**Affiliations:** 1 Department of Microbiology, Parasitology and Virology, Faculty of Nursing, Carol Davila University of Medicine and Pharmacy, 020021 Bucharest, Romania; loredana.manolescu@umfcd.ro; 2 Clinical Laboratory of Medical Microbiology, Marius Nasta Institute of Pneumology, 050159 Bucharest, Romania; 3 National Reference Laboratory of Tuberculosis, Marius Nasta Institute of Pneumology, 050159 Bucharest, Romania; oana.popescu@marius-nasta.ro; 4 Cardiothoracic Department, Faculty of Medicine, Carol Davila University of Medicine and Pharmacy, 020956 Bucharest, Romania; beatrice.mahler@umfcd.ro; 5 1st Pneumology Department, Marius Nasta Institute of Pneumology, 050159 Bucharest, Romania; 6 3rd Pulmonology Section, 4th Department—Cardio-Thoracic Pathology, Carol Davila University of Medicine and Pharmacy, 020021 Bucharest, Romania; dragos.zaharia@umfcd.ro; 7 7th Pulmonology Department, Marius Nasta Institute of Pneumology, 050159 Bucharest, Romania

**Keywords:** *Klebsiella pneumoniae*, antimicrobial resistance, PDR, carbapenem resistance, colistin resistance, ICU, Romania

## Abstract

**Background:** *Klebsiella pneumoniae* is a leading cause of healthcare-associated infections and a major vehicle for the dissemination of antimicrobial resistance (AMR). Romania ranks among the most affected European countries for carbapenem-resistant *K. pneumoniae* (CRKP), yet data from specialized pulmonology–critical care settings remain limited. **Objective:** This study aimed to characterize the antimicrobial resistance profiles of *K. pneumoniae* clinical isolates—including MDR/XDR/PDR classification, carbapenem and colistin resistance rates, specimen and ward distribution, and category-specific resistance patterns—at a tertiary pulmonology–critical care institution in Bucharest, Romania. **Methods:** A retrospective, single-centre analysis of 203 *K. pneumoniae* clinical isolates from critical patients, which were collected at the Institute of Pneumology Marius Nasta, Bucharest, between July 2021 and May 2026. Susceptibility testing was performed using the VITEK^®^2 system (bioMérieux), with results interpreted per EUCAST breakpoints. **Results:** Of 203 isolates, 112 (55.2%) were PDR, 36 (17.7%) XDR, six (3.0%) MDR, and 49 (24.1%) non-MDR. Carbapenem resistance was present in 134 isolates (66.0%); colistin resistance was present in 49 of 73 isolates tested (67.1%). Bronchial/tracheal aspirate was the dominant specimen source (61.1%), and intensive care units accounted for 61.1% of isolates. Resistance rates exceeded 75% for monobactams, first/second-generation cephalosporins, and fluoroquinolones. **Conclusions:** This cohort demonstrates severe AMR burden in this setting, consistent with the most concerning reports for *K. pneumoniae* in the literature. The findings underscore the urgent need for enhanced antimicrobial stewardship, strengthened infection control in ICU settings, and molecular surveillance of carbapenemase-producing high-risk clonal lineages in Romanian tertiary care.

## 1. Introduction

*Klebsiella pneumoniae* is a Gram-negative, encapsulated, facultative anaerobic member of the Enterobacterales order that asymptomatically colonizes the human gastrointestinal tract and oropharynx but is also one of the leading causes of healthcare-associated infections worldwide, including ventilator-associated pneumonia, bloodstream infections, urinary tract infections, and intra-abdominal infections [1]. Over the past two decades, *K. pneumoniae* has evolved from a manageable opportunistic pathogen into one of the most formidable agents of antimicrobial resistance (AMR), owing to its remarkable genomic plasticity and its capacity to acquire and disseminate resistance determinants through plasmids, transposons, and other mobile genetic elements [1,2]. High-risk clonal lineages of multidrug-resistant (MDR) and extensively drug-resistant (XDR) *K. pneumoniae* have repeatedly demonstrated the ability to cause multicontinent outbreaks, cementing the species’ status as a major global vehicle for the dissemination of antibiotic resistance [1,3].

The scale of this threat is reflected in recent global burden estimates: in 2019 alone, bacterial AMR was associated with an estimated 4.95 million deaths worldwide, including 1.27 million deaths directly attributable to resistant infections, with *K. pneumoniae* among the leading contributing pathogens [4]. In recognition of this threat, the 2024 World Health Organization Bacterial Priority Pathogens List ranked carbapenem-resistant *K. pneumoniae* as the single highest-scoring pathogen among 24 antibiotic-resistant bacteria evaluated, ahead of carbapenem-resistant *Acinetobacter baumannii* and rifampicin-resistant *Mycobacterium tuberculosis*, underscoring the urgency of continued surveillance and the development of novel therapeutic strategies [5].

To enable consistent characterization of resistance phenotypes across studies, Magiorakos et al. proposed standardized interim definitions classifying isolates as multidrug-resistant (MDR; acquired non-susceptibility to at least one agent in three or more antimicrobial categories), extensively drug-resistant (XDR; susceptible to agents in only one or two categories), or pandrug-resistant (PDR; non-susceptible to all agents in all tested categories) [6]. These definitions, now widely adopted in the international literature, were applied throughout the present study.

Carbapenem-resistant *K. pneumoniae* (CRKP) has become endemic in several European countries [7]. According to the 2023 EARS-Net report, the EU/EEA population-weighted mean rate of carbapenem resistance among invasive *K. pneumoniae* isolates was 13.3%, with the estimated EU incidence of CRKP bloodstream infections reaching 3.97 cases per 100,000 population in 2023 (country range 0.00–21.44)—a 57.5% increase from the 2019 baseline and above the EU 2030 target of 2.39 cases per 100,000 [8]. The 2024 ECDC Surveillance Atlas of Infectious Diseases ranked Romania as the third most affected European country, with a resistance rate of 50.3%, surpassed only by Greece (60.2%) and Bulgaria (67.6%) [9]. Within Romania, single-centre studies have documented a sharp rise in CRKP prevalence—from 4.9% in 2010 to over 40% by 2024—driven largely by the spread of KPC, NDM, and OXA-48-type enzymes, increasingly as dual NDM + OXA-48-type producers [10,11].

The progressive loss of carbapenem efficacy has driven renewed reliance on colistin as a last-resort agent for carbapenem-resistant Gram-negative infections [12]. This increased clinical dependence has been accompanied by a corresponding rise in colistin resistance: a 2022 systematic review reported a global pooled colistin resistance prevalence of 3.1% overall, rising to 12.9% among isolates from 2020 onward [13]. In the Balkan region, colistin-resistant CRKP has emerged as a particular concern, exemplified by an intra-hospital outbreak at a Sofia, Bulgaria ICU in which colistin-resistant strains co-harbouring *bla*NDM-5 and *bla*OXA-232 emerged following a near fourfold increase in institutional colistin consumption [14,15]. A comparable dynamic has been described in Romania, where colistin consumption rose by 186% between 2019 and 2024, and colistin susceptibility among NDM + OXA-48-type producers fell to only 16.5% [10].

Despite this growing body of evidence, data remain comparatively limited on the resistance burden of *K. pneumoniae* in Romanian pulmonology and respiratory critical care settings specifically, where prolonged mechanical ventilation, repeated antibiotic exposure, and a nationally referred patient population may create particularly strong selective pressure for the emergence and persistence of MDR, XDR, and PDR phenotypes. The present study therefore aimed to characterize the antimicrobial resistance profiles—including MDR/XDR/PDR classification, carbapenem and colistin resistance rates, specimen and ward distribution, and category-specific resistance patterns—of 203 *K. pneumoniae* clinical isolates collected over a nearly five-year period at a national pulmonology referral institution in Bucharest, and to situate these findings within the broader Romanian, Balkan, and European epidemiological context.

## 2. Results

### 2.1. Antimicrobial Resistance Profiles and MDR/XDR/PDR Classification

**Figure 1:**
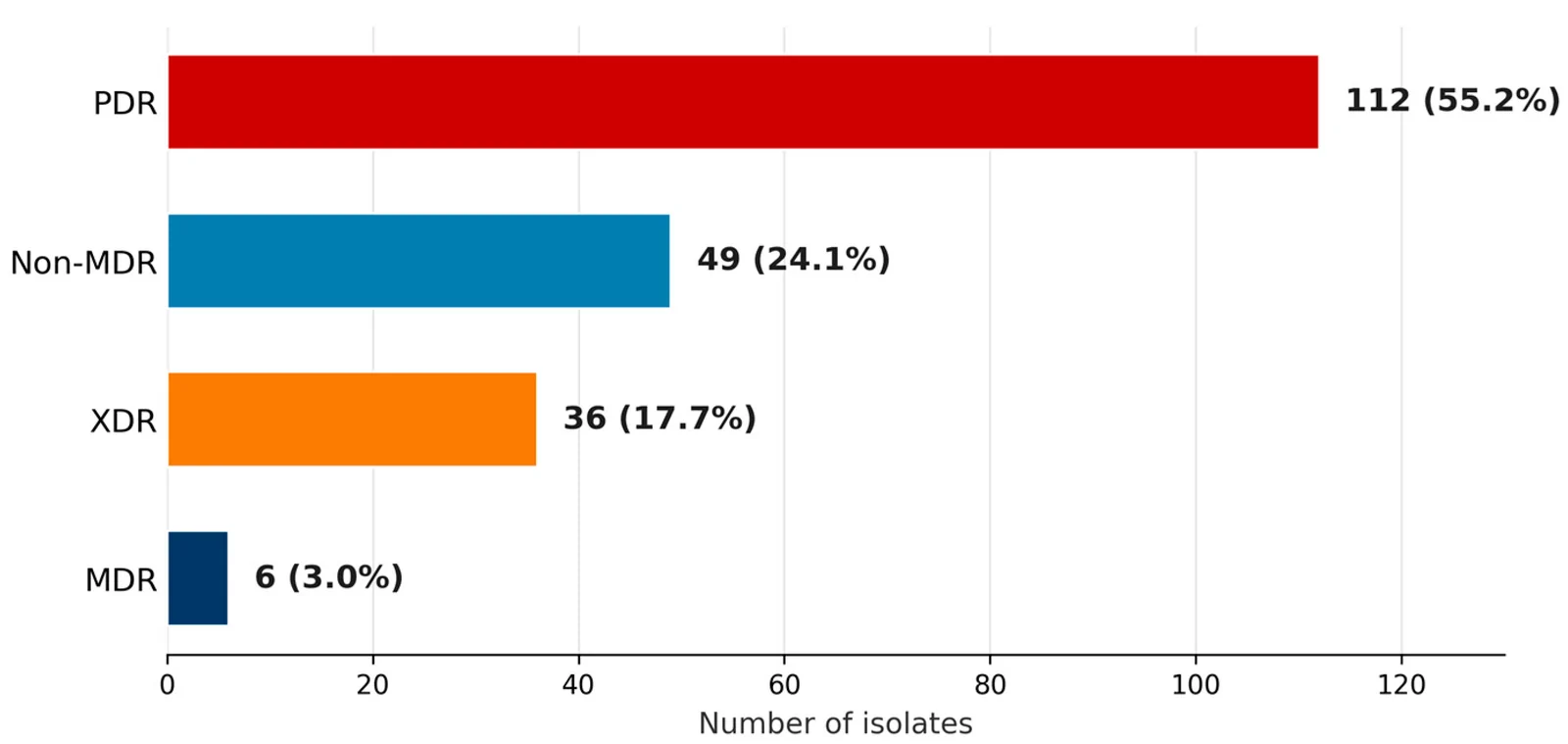
Distribution of resistance phenotypes (MDR, XDR, PDR, non-MDR) among 203 *Klebsiella pneumoniae* clinical isolates from critical-care patients at the Institute of Pneumology “Marius Nasta”, Bucharest, Romania (July 2021–May 2026). MDR was defined as non-susceptibility to at least one agent in three or more antimicrobial categories; XDR as non-susceptibility in all but one or two categories; and PDR as non-susceptibility to all agents in all antimicrobial categories tested, according to the standardized definitions of Magiorakos et al. [6].

The overall resistance burden was markedly severe, with the majority of isolates classified as PDR or XDR (Figure 1). Combined, PDR and XDR accounted for 73% of all isolates, reflecting an exceptionally advanced resistance phenotype distribution. These findings were due to the fact that we performed automated identification and antibiotic sensitivity testing on all isolates that appeared as MDR in standard antibiotic sensitivity testing.

**Figure 2:**
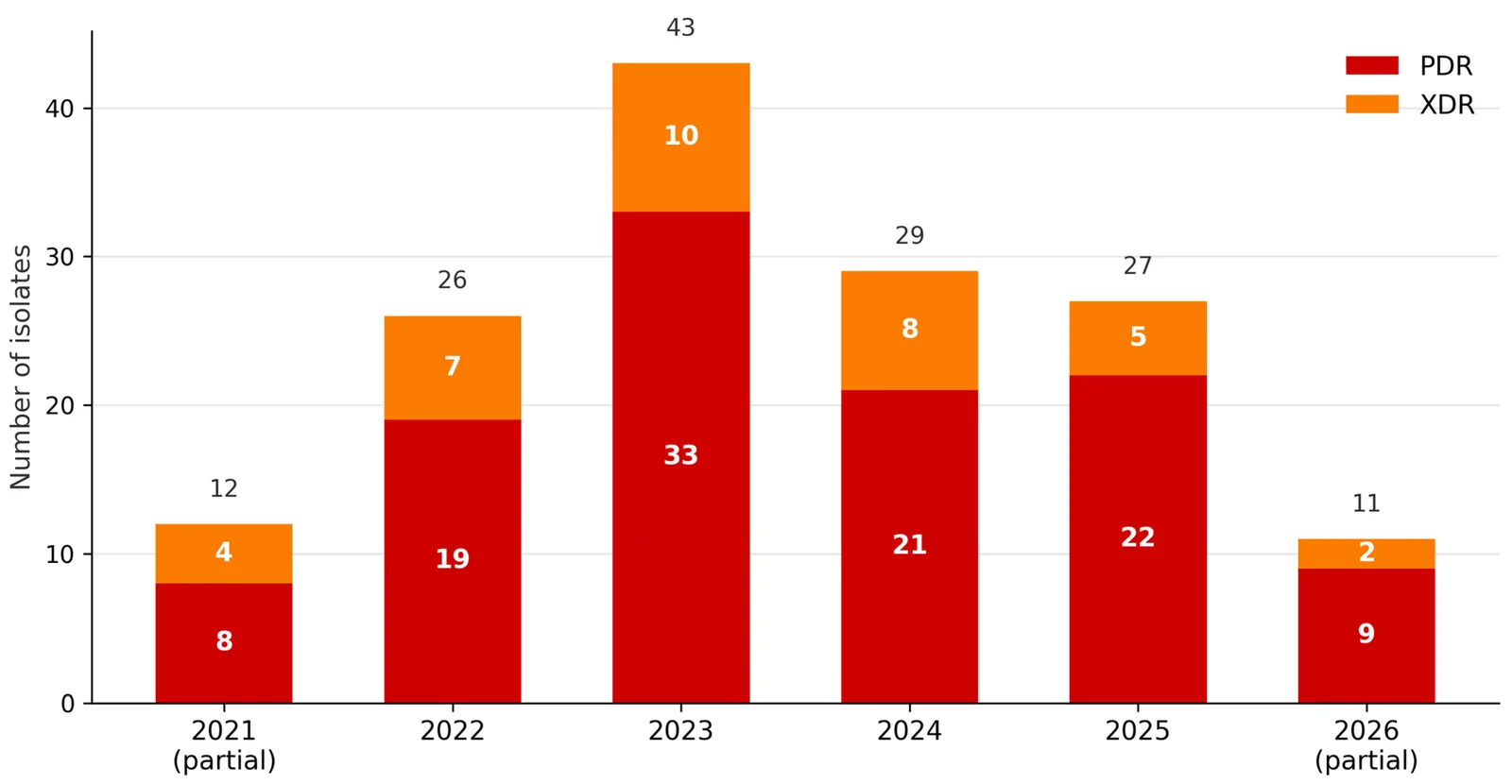
Temporal Distribution of PDR and XDR *K. pneumoniae* isolates by year (2021–2026). Data collection began in July 2021 and ended in May 2026; the 2021 and 2026 bars therefore represent partial-year data (July–December 2021 and January–May 2026, respectively), while 2022–2025 represent complete calendar years.

Temporally, PDR isolates were identified across all years from 2021 onward, with counts peaking in 2022–2023 and remaining persistently elevated through 2024–2025 (Figure 2).

### 2.2. Carbapenem and Colistin Resistance

Carbapenem resistance was identified in 134 of 203 isolates (66.0%), representing approximately two-thirds of the cohort. Colistin resistance was documented in 49 of 73 isolates tested (67.1%), representing a major clinical concern given colistin’s role as a last-resort antimicrobial agent.

### 2.3. Carbapenemases Production

A positive result for both OXA-48 and NDM on the RESIST-5 O.K.N.V.I. assay was detected in 112 (55.2%) isolates and was interpreted as evidence of co-production of OXA-48-like and NDM carbapenemases by the isolates, indicating the presence of multiple acquired carbapenem resistance mechanisms. The temporal distribution of ESBL positivity among tested isolates is shown in Supplementary Figure S1.

Detection of both OXA-48 and NDM in the RESIST-5 O.K.N.V.I. test means that the bacterial isolate carries two different carbapenemase enzymes simultaneously: OXA-48-like carbapenemase, a class D β-lactamase that hydrolyzes carbapenem antibiotics and is commonly found in Enterobacterales, and NDM (New Delhi metallo-β-lactamase), a class B metallo-β-lactamase that confers resistance to most β-lactam antibiotics, including carbapenems.

The presence of both enzymes indicates a co-producing multidrug-resistant organism, which is associated with: high-level resistance to carbapenems; limited therapeutic options; increased potential for transmission due to plasmid-mediated resistance genes and important infection prevention and control implications.

### 2.4. Specimen Source and Clinical Distribution

**Figure 3:**
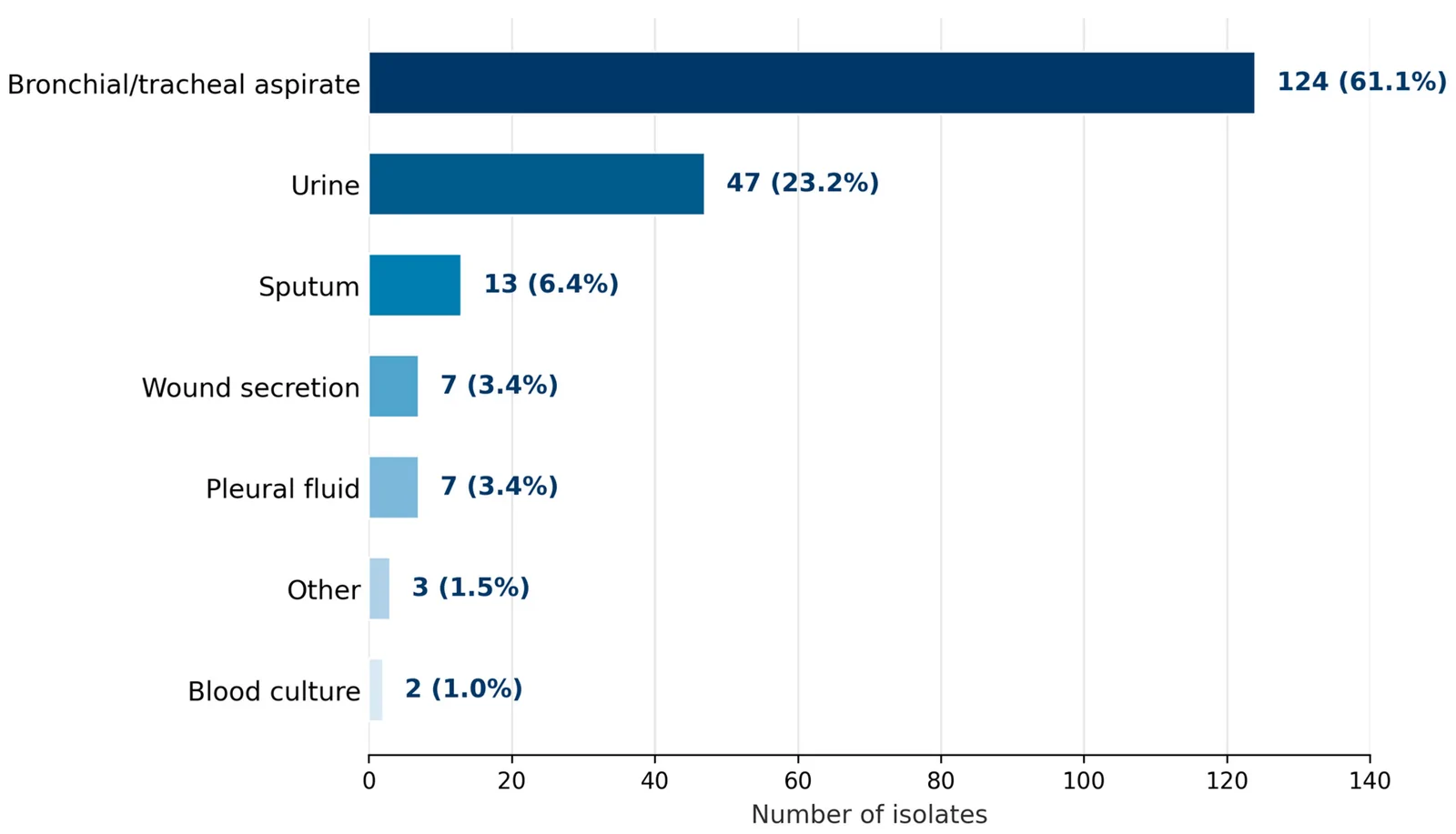
Distribution of isolates by specimen source (*n* = 203).

The predominant specimen source was bronchial/tracheal aspirate, accounting for more than 60% of isolates, followed by urine and, less frequently, sputum, pleural fluid, wound secretion, and other sources (Figure 3).

The ICU/ATI was the most frequently implicated ward (*n* = 124, 61.1%), followed by general pulmonology wards (*n* = 57, 28.1%); the remaining isolates (*n* = 22, 10.8%) originated from surgical wards, the outpatient clinic, and other specialty or referral units. This distribution is consistent with the published literature characterizing respiratory tract specimens and ICU origin as the dominant source for drug-resistant *K. pneumoniae* [16,17,18].

### 2.5. Category-Specific Resistance Rates

**Figure 4:**
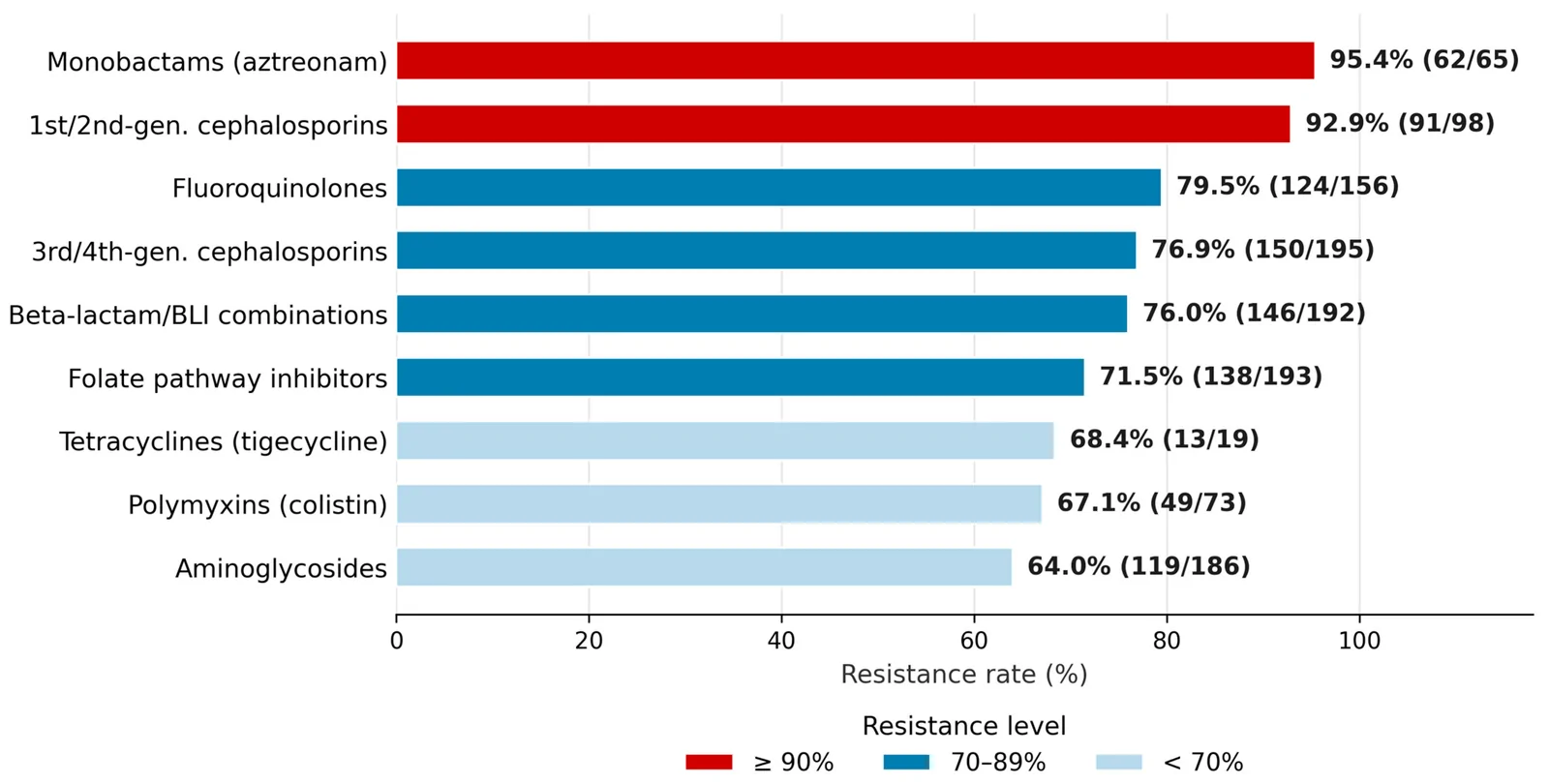
Category-specific antimicrobial resistance rates among 203 *Klebsiella pneumoniae* isolates. Antimicrobial categories comprised: beta-lactam/beta-lactamase inhibitor combinations (amoxicillin/clavulanic acid, ampicillin/sulbactam, piperacillin/tazobactam); first/second-generation cephalosporins (cefuroxime, cefuroxime axetil); third/fourth-generation cephalosporins (cefotaxime, ceftazidime, ceftriaxone, cefepime); monobactams (aztreonam); aminoglycosides (gentamicin, tobramycin, amikacin); fluoroquinolones (ciprofloxacin, levofloxacin, moxifloxacin); folate pathway inhibitors (trimethoprim/sulfamethoxazole); tetracyclines (tigecycline); and polymyxins (colistin).

**Figure 5:**
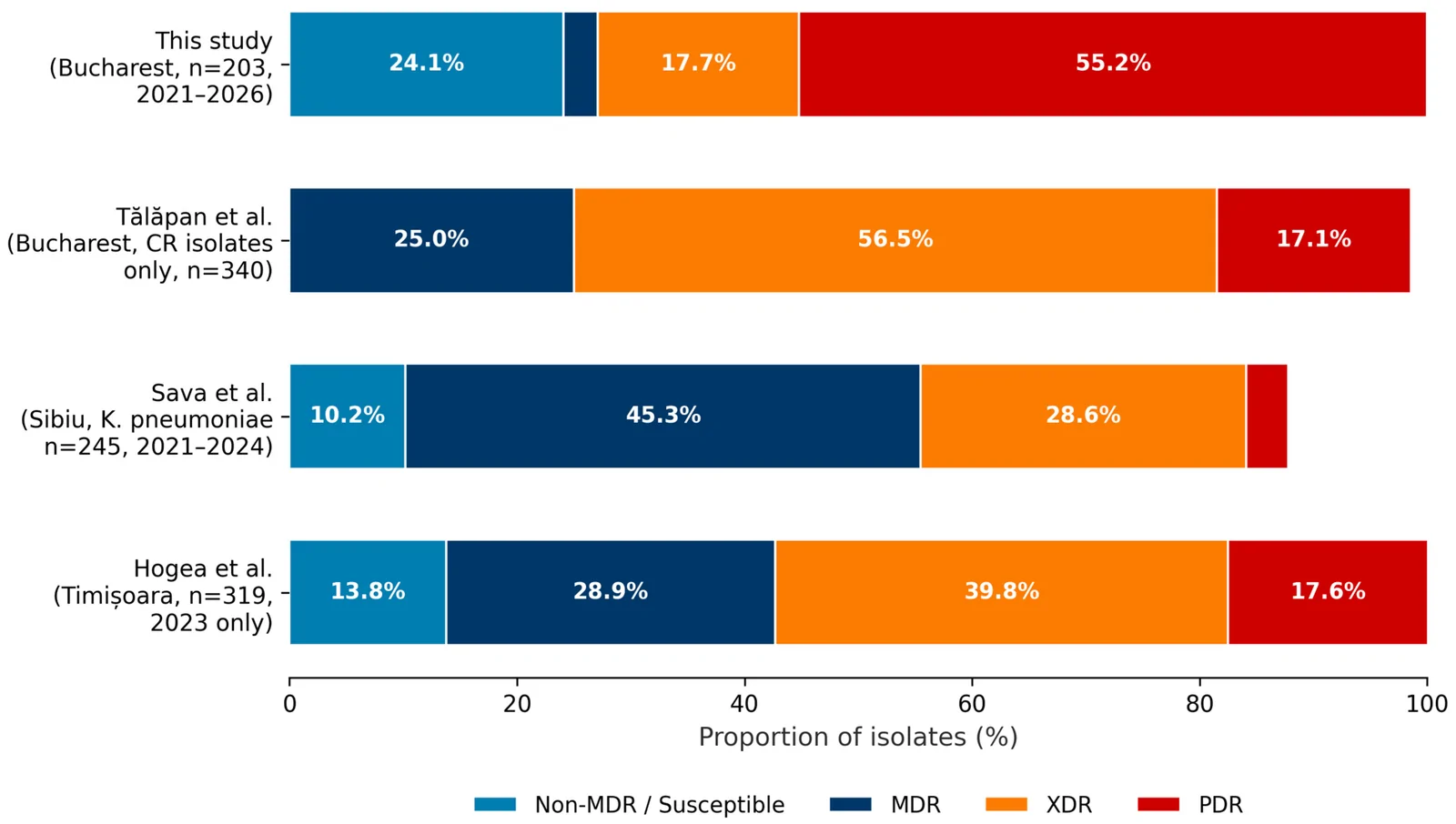
Distribution of MDR/XDR/PDR phenotypes in the present cohort compared with other published Romanian *K. pneumoniae* series [9,19,20]. Note: the Tălăpan et al. comparator reflects the subset of carbapenem-resistant isolates only (no non-MDR category applicable), whereas the other three cohorts reflect their complete respective study populations.

Resistance across individual antibiotic categories was consistently high, though it varied in magnitude, ranging from 63.9% (aminoglycosides) to 95.4% (monobactams/aztreonam); monobactams, and first/second-generation cephalosporins each exceeded 90% (Figure 4). Tetracycline-class resistance (tigecycline) was notably lower in the tested denominator (*n* = 19), warranting cautious interpretation. (Figure 5). A more granular breakdown of resistance by individual antimicrobial agent is provided in Supplementary Figure S2.

## 3. Discussion

This study describes the antimicrobial resistance profile of 203 *Klebsiella pneumoniae* clinical isolates collected over a nearly five-year period at a tertiary pulmonology–critical care institution in Bucharest. The findings reveal an exceptionally severe resistance burden, with PDR and XDR phenotypes together accounting for 73% of isolates, a carbapenem resistance rate of 66.0%, a dual OXA-48/NDM carbapenemase co-production rate of 55.2% and colistin resistance affecting more than two-thirds of tested isolates (49/73, 67.1%). These figures place our cohort among the most resistant *K. pneumoniae* populations reported to date in the European literature.

### 3.1. MDR/XDR/PDR Distribution

The predominance of PDR isolates (55.2%), and also carbapenemase producer isolates, contrasts sharply with resistance phenotype distributions reported in other Romanian studies. While a two-year retrospective study at a Romanian tertiary hospital found XDR to be the dominant phenotype among carbapenem-resistant isolates with PDR representing a comparatively smaller share [9], our cohort shows the inverse pattern, with PDR isolates outnumbering XDR by more than three to one (Figure 5).

Likewise, the four-year ICU surveillance reported by Sava et al. described an evolving trajectory in which MDR phenotypes dominated early in the study period and were progressively replaced by XDR and PDR strains [17]. A comparable inverse pattern—moderate PDR relative to MDR/XDR—has also been reported outside Bucharest. In a four-year ESKAPE surveillance study at a Sibiu ICU, *K. pneumoniae* accounted for 245 of 573 isolates (42.8%), with an MDR:XDR:PDR distribution of 45.3%:28.6%:3.7%—closer to the “classic” resistance escalation pattern than to the PDR-dominant profile observed in our cohort [19]. By contrast, a six-year surveillance study from a tertiary centree in Timișoara documented a trajectory more consistent with our findings: XDR and PDR strains rose steadily from 30.5% combined in 2018 to 57.4% in 2023, with PDR alone reaching 17.6% by the final year [20]. Taken together, these regionally diverse Romanian datasets suggest that the severe, PDR-dominant resistance burden observed at our institution is not confined to Bucharest, though its magnitude varies considerably by setting and patient case-mix. Our data, by comparison, show PDR isolates present at consistently high levels from the earliest study years onward, suggesting that the resistance ecology at our institution may have already reached an advanced, relatively stable plateau before the start of data collection. This could plausibly reflect the referral function of the institution as a national pulmonology center, concentrating patients with prior prolonged hospitalization, repeated antibiotic exposure, and pre-existing colonization with highly resistant organisms before admission [21].

### 3.2. Carbapenem and Colistin Resistance Evaluation

**Figure 6:**
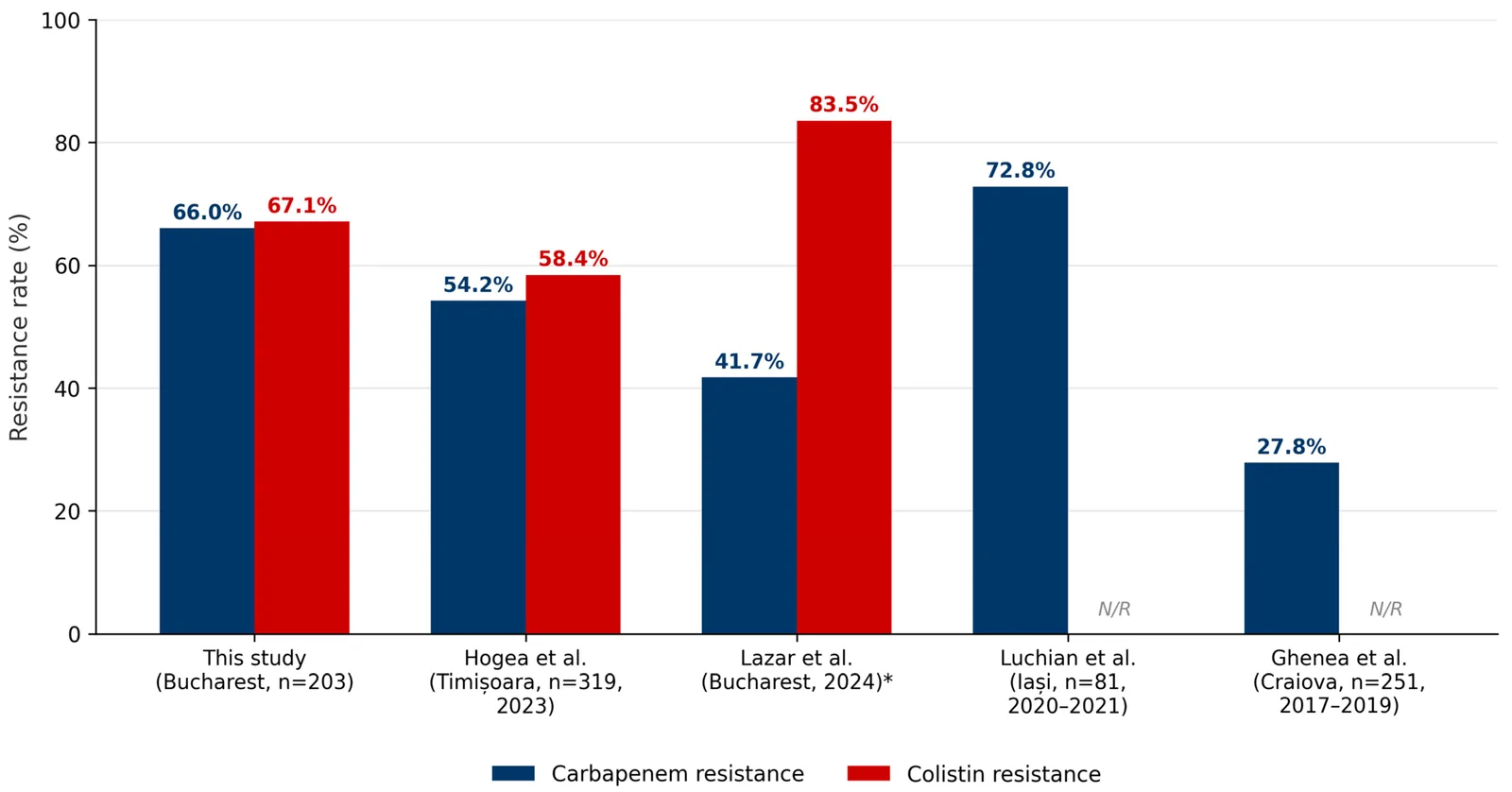
Carbapenem and colistin resistance rates in the present cohort compared with other published Romanian *K. pneumoniae* series [10,20,22,23]. * Colistin resistance for Lazar et al. reflects the subgroup of NDM + OXA-48-type dual carbapenemase producers only, not the overall cohort, and is not directly comparable to the other bars. N/R = not reported in the original publication.

The carbapenem resistance rate observed here (66.0%) considerably exceeds both the EU/EEA population-weighted average [8] and the rate reported for Romania as a whole in the most recent ECDC Surveillance Atlas [9], where Romania already ranked third among European countries. Regional data outside the capital similarly document a marked escalation in carbapenem and colistin resistance. In Timișoara, carbapenem resistance (ertapenem) rose from 35.6% in 2018 to 54.2% in 2023, while colistin resistance increased dramatically from 10.2% to 58.4% over the same period, and carbapenemase-positive isolates rose from 15.3% to 51.7%, driven predominantly by OXA-48-like enzymes (7.6% to 31.3%) [20]. These trends parallel, and in the case of colistin resistance considerably exceed, those observed in our cohort, reinforcing that the escalating carbapenem–colistin resistance axis is a broader national phenomenon rather than a Bucharest-specific finding (Figure 6).

Our rate is, however, closely comparable to that reported from a Turkish tertiary ICU-heavy cohort with a similar specimen and ward distribution [18,24], reinforcing the idea that case-mix—specifically the concentration of mechanically ventilated, ICU-admitted patients—is a major driver of carbapenem resistance prevalence at the institutional level, independent of national averages.

The colistin resistance rate of 67.1% (49/73 isolates tested) is particularly concerning, given colistin’s role as a last-resort agent for carbapenem-resistant Gram-negative infections. This figure is approximately 21-fold higher than global pooled estimates from bloodstream infections (3.1%) [13] and roughly six-fold higher than the ICU-specific global estimate (11.5%). The documented 186% rise in colistin consumption in Romania between 2019 and 2024 [10] offers a plausible mechanistic explanation, consistent with the selective-pressure dynamics described in the Bulgarian outbreak report [14,15] and with the comparably low colistin susceptibility (16.5%) reported among NDM/OXA-48-type producers at another Romanian referral centre [10]. Taken together, these findings suggest that colistin resistance in Romanian tertiary care settings may no longer be a sporadic event but an emerging regional pattern tied to intensifying last-line antibiotic use in response to carbapenem resistance—a feedback loop with serious implications for future treatment options.

### 3.3. Specimen Source and Clinical Setting

The overwhelming predominance of bronchial/tracheal aspirates (61.1%) and ICU origin (61.1%) is consistent with the expected epidemiology of a pulmonology–critical care referral centre and mirrors findings from comparable cohorts, including both the Romanian ICU series by Sava et al. [17] and the Turkish multicentre study [24]. This concordance supports the interpretation that the resistance burden observed is closely linked to the population served—patients with chronic respiratory pathology, frequent mechanical ventilation, and prolonged ICU stay—rather than representing a generalized phenomenon across all clinical settings [25]. The low proportion of bloodstream isolates (1.0%) limits direct comparability with studies focused on invasive/bacteraemia infections, such as the EARS-Net and global pooled colistin-resistance estimates.

The contrast with less severely affected populations further supports a case-mix-driven interpretation. A pediatric cohort from Constanța found *K. pneumoniae* in only 19.35% of positive clinical isolates, with resistance concentrated in ceftriaxone (50%) and ampicillin (44%), while carbapenems and amikacin remained largely effective [26]—a markedly milder resistance profile than observed in our ICU-referral cohort. This gradient, from a general pediatric population in Constanța through the mixed ICU/ward cohort in Sibiu to the severely affected referral population described here, suggests that patient case-mix and level of care, rather than geography alone, are major determinants of the resistance burden observed across Romanian centres.

Data from north-eastern Romania provide a further regional comparator with a notably similar ward distribution. In a descriptive study of 81 *K. pneumoniae* isolates causing healthcare-associated infections at a neurosurgical hospital in Iași (2020–2021), the ICU accounted for 65.4% of cases [22]—closely matching the 61.1% ICU/ATI proportion observed in our cohort—alongside high rates of ESBL production (81.5%) and carbapenem resistance (72.8%). By contrast, an earlier (2017–2019) study of 251 *Klebsiella* spp. isolates from a general county hospital in Craiova reported considerably lower resistance to meropenem (27.8%), ciprofloxacin (30.6%), and amikacin (13.5%) [23], a population drawn predominantly from general wards rather than critical care. Together with the Constanța pediatric data, these findings reinforce that ICU/referral case-mix, rather than region, is the principal driver of the elevated resistance burden reported here.

Consistent with this, resistance rates for key antimicrobials were substantially higher among ICU/ATI isolates than among those from other wards (Supplementary Figure S3). Interestingly, resistance to colistin, meropenem, and ciprofloxacin was numerically higher among urine isolates than among bronchial/tracheal aspirates (Supplementary Figure S4), a pattern that warrants further investigation given the smaller sample size for non-respiratory specimens.

### 3.4. Category-Specific Resistance and Clonal Considerations

Resistance rates across nearly all antibiotic classes exceeded those reported in comparable meta-analyses and regional surveillance data, often substantially. These rates broadly exceed those reported in comparable meta-analyses and regional surveillance data. Fluoroquinolone resistance in *K. pneumoniae* from bloodstream infections has been reported at 45.3% globally [13], while aminoglycoside resistance (gentamicin) was 33.3% in the same meta-analysis. The substantially higher rates observed in our cohort are consistent with the ICU-dominated, ventilator-associated infection context and the endemic spread of high-risk clonal lineages—particularly NDM + OXA-48-type dual carbapenemase producers—increasingly documented across Romanian tertiary care centres [11].

This pattern of broad, near-universal resistance across structurally unrelated antibiotic classes raises the hypothesis that dissemination of high-risk clonal lineages co-harbouring multiple resistance determinants, rather than independent accumulation of resistance through sequential selective pressure, may be the predominant driver in this cohort—though, in the absence of molecular typing data, this interpretation remains speculative and requires confirmation [27]. The repeated documentation of NDM + OXA-48-type dual carbapenemase producers as an emerging and dominant genotype in Romanian tertiary centres [11] is consistent with this interpretation, although molecular confirmation of carbapenemase genes and clonal typing (e.g., MLST, capsular typing) were beyond the scope of the present phenotypic study [28]. The frequent detection of isolates co-harbouring OXA-48-like and NDM carbapenemases is noteworthy, particularly in light of the recent identification in Romania of the high-risk ST383 *Klebsiella pneumoniae* clone carrying *bla*NDM-5 and *bla*OXA-48. Although sequence typing was not performed in the present study, the predominance of this carbapenemase profile suggests that similar ST383-related strains may also be circulating in our institution. Further genomic investigations are required to confirm their presence and evaluate their epidemiological impact.

### 3.5. Limitations

Several limitations should be acknowledged. First, this is a single-centre, retrospective study, which limits generalizability. Second, the absence of molecular characterization (carbapenemase gene typing, clonal lineage analysis) precludes definitive conclusions about resistance mechanisms and clonal relatedness. Third, the number of isolates tested varied across antibiotic categories, which may affect the precision of category-specific resistance estimates. Additionally, colistin susceptibility was determined using VITEK^®^2 alone, without confirmatory broth microdilution, which EUCAST and CLSI have specified since 2016 as the only validated reference method for colistin AST. Automated systems, including VITEK^®^2, have been shown in independent validation studies to have unacceptably high, very major error rates for colistin—i.e., a tendency to misclassify truly resistant isolates as susceptible—meaning the colistin resistance rate reported here (20.7%) should be interpreted with caution and may not precisely reflect the true prevalence of colistin resistance in this cohort. Furthermore, colistin susceptibility testing was performed on only 73 of 203 isolates (36%), reflecting variation in routine panel composition over the study period (Section 2.2); the reported colistin resistance rate (67.1%) should therefore be interpreted as representative of the tested subset rather than the full cohort. Additionally, the RESIST-5 O.K.N.V.I. assay identified dual OXA-48/NDM co-production in 55.2% of isolates; however, single-carbapenemase-positive isolates (OXA-48-like, NDM, KPC, VIM, or IMP alone) and non-enzymatic mechanisms (e.g., porin loss, efflux) were not separately characterized in the remaining carbapenem-resistant isolates, limiting full mechanistic resolution of the 66.0% overall carbapenem resistance rate. Furthermore, carbapenemase characterization relied solely on the RESIST-5 O.K.N.V.I. assay, a rapid immunochromatographic lateral-flow test detecting five carbapenemase families (OXA-48-like, KPC, NDM, VIM, IMP) at the protein level; no molecular confirmation (e.g., PCR or whole-genome sequencing of the corresponding *bla* genes) was performed. While RESIST-5 has demonstrated good performance in validation studies, protein-level detection cannot exclude rare or novel carbapenemase variants not covered by the assay’s antibody panel, nor can it substitute gene-level confirmation when definitive molecular characterization is required. The most important limitation comes from the fact that the presented isolates were only isolates selected from ICU patients or critical patients waiting to be admitted into the ICU. All isolates tested initially MDR in standard antibiotic sensitivity testing. Finally, possible duplicate isolates from the same patient over time were not explicitly addressed, which could inflate resistance estimates if recurrent colonization occurred.

## 4. Materials and Methods

### 4.1. Study Design and Setting

This was a retrospective, single-centre observational study conducted at the Institute of Pneumology “Marius Nasta”, a national pulmonology and respiratory critical care referral institution in Bucharest, Romania. All non-duplicate clinical isolates of *Klebsiella pneumoniae* recovered from routine diagnostic specimens identified on VITEK^®^2 automated system (bioMérieux, Marcy-l’Étoile, France) between July 2021 and May 2026 were eligible for inclusion. The isolates came from patients admitted to the intensive care unit (ICU), or from patients in general wards who were awaiting ICU bed availability on the basis of at least one of the following: a documented severity score (SOFA, qSOFA, or NEWS2) indicating critical illness; a clinical need for respiratory support (high-flow oxygen or non-invasive ventilation) or vasopressor support; or inclusion on the hospital’s ICU waiting list regardless of the specific triggering criterion. All isolates included into the study tested initially MDR in standard AST (antibiotic sensitivity testing). A total of 203 isolates met inclusion criteria and were retained for analysis.

### 4.2. Bacterial Identification and Antimicrobial Susceptibility Testing

Primary isolation was performed on blood agar and CLED agar for all specimens, with the addition of chocolate agar for respiratory specimens. Species identification was performed using the VITEK^®^2 Gram-Negative Identification (ID-GN) card, and antimicrobial susceptibility testing was performed using VITEK^®^2 AST-GN cards, following inoculum standardization to a 0.5 McFarland suspension per the manufacturer’s standard protocol (bioMérieux, Marcy-l’Étoile, France). During the study period, card configurations included AST-N437 (an ESBL-confirmation card typically used for outpatient and pediatric isolates), AST-N439 (designed for multidrug-resistant Gram-negative bacilli), and the AST-N438/AST-XN26 combination (an extended-range configuration including additional agents such as eravacycline), reflecting the laboratory’s routine practice of pairing a base card with a complementary extended card for isolates requiring a broader panel [29]. Card configurations evolved over the five-year study period as antimicrobial agent availability and testing protocols were updated; consequently, not every antimicrobial category was tested for every isolate, and the denominator of isolates tested is reported alongside each category-specific resistance rate [15,16]. Species identification and AST, including colistin, were performed using VITEK^®^2 alone; no confirmatory testing by MALDI-TOF mass spectrometry, molecular methods, or broth microdilution was performed. Antimicrobial agents were grouped into the following categories: first/second-generation cephalosporins, third/fourth-generation cephalosporins, beta-lactam/beta-lactamase inhibitor combinations, monobactams (aztreonam), carbapenems, aminoglycosides, fluoroquinolones, folate pathway inhibitors (trimethoprim/sulfamethoxazole), tetracyclines (tigecycline), and polymyxins (colistin). Not every category was tested for every isolate, owing to variation in routine panel composition over the study period; the denominator of isolates tested is reported alongside each category-specific resistance rate.

### 4.3. Resistance Phenotype Classification

Isolates were classified as MDR, XDR, PDR, or non-MDR according to the standardized interim definitions proposed by Magiorakos et al. on behalf of the ECDC and CDC [6]. Briefly, MDR was defined as acquired non-susceptibility to at least one agent in three or more antimicrobial categories; XDR as non-susceptibility in all but one or two categories; and PDR as non-susceptibility to all agents in all antimicrobial categories tested. Carbapenem resistance and colistin resistance were additionally analyzed as standalone phenotypic outcomes, given their particular clinical and epidemiological significance. Carbapenemase production was assessed using the RESIST-5 O.K.N.V.I. assay (CORIS BioConcept, Gembloux, Belgium), a rapid immunochromatographic lateral-flow test that qualitatively detects OXA-48-like, KPC, NDM, VIM, and IMP carbapenemases from bacterial isolates [30].

### 4.4. Data Collection

For each isolate, the following data were extracted from laboratory records: date of isolation, specimen source (bronchial/tracheal aspirate, urine, sputum, pleural fluid, wound secretion, or blood culture), hospital ward of origin, intensive care unit (ICU) versus general ward, and the full antimicrobial susceptibility profile across all tested agents. Only the first isolate per patient per resistance phenotype was considered where applicable, consistent with standard practice in comparable surveillance studies [5].

### 4.5. Statistical Analysis

Descriptive statistics were used to summarize the distribution of resistance phenotypes, carbapenem and colistin resistance rates, specimen source, ward distribution, and category-specific resistance rates. Results are expressed as absolute numbers and percentages, with the tested denominator specified for each antibiotic category. Temporal distribution of PDR isolates was summarized by calendar year. No formal inferential statistical testing was performed; comparisons with published regional and global data were made descriptively.

### 4.6. Ethical Considerations

Given the retrospective, laboratory-data-based nature of the study, no direct patient contact or additional clinical intervention was involved. Data were derived from routine diagnostic microbiology records and analyzed in de-identified form, without access to patient-identifying information. All patients provided general written consent at hospital admission for the use of their clinical and laboratory data for research and educational purposes, in accordance with standard practice at the Institute of Pneumology “Marius Nasta”. For the present study specifically, the Institutional Ethics Committee reviewed the retrospective, de-identified nature of the dataset and waived the requirement for additional individual informed consent (approval no. 9180/13.05.2025).

## 5. Conclusions

This study documents an alarmingly high and sustained burden of multidrug, extensive, and pan-drug resistance among *K. pneumoniae* isolates in a Romanian pulmonology–critical care referral centre, the high co-occurrence of OXA-48 and NDM carbapenemase in associations with extensive antimicrobial resistance and limited therapeutic options.

These findings underscore the urgent need for enhanced antimicrobial stewardship, strengthened infection control measures in ICU settings, and molecular surveillance to characterize the carbapenemase genotypes and clonal lineages driving this resistance trajectory. Given the parallel colistin resistance rate of 67.1% among tested isolates, the therapeutic options for infections caused by these organisms are becoming critically limited, reinforcing the importance of novel antimicrobial agents and combination regimens in this setting.

## Data Availability

The data presented in this study are available on request from the corresponding author due to privacy restrictions.

## Supplementary Materials

The following supporting information can be downloaded at: https://www.mdpi.com/article/10.3390/antibiotics15080774/s1, Figure S1: ESBL positivity among *Klebsiella pneumoniae* isolates tested for ESBL production, by year of isolation (2021–2026). Bars show the number of isolates tested per year (light blue) and the ESBL-positive subset (dark red), with the positive fraction and percentage labeled above each year. Data collection began in July 2021 and ended in May 2026; the 2021 and 2026 columns therefore reflect partial-year data; Figure S2: Resistance rate by individual antimicrobial agent among *Klebsiella pneumoniae* isolates (*n* ≥ 15 tested per agent). Cells show the percentage of isolates classified as resistant among those tested, with the corresponding fraction (resistant/tested) in parentheses; color intensity scales with resistance rate (0–100%); Figure S3: Resistance rates for six key antimicrobial agents, stratified by hospital ward (ICU/ATI vs. other wards). Bars show the percentage of isolates resistant among those tested, with the resistant/tested fraction labeled above each bar; Figure S4: Resistance rates for colistin, meropenem, and ciprofloxacin, stratified by specimen source, restricted to specimen types with at least five isolates tested per agent. Fractions above each bar indicate resistant/tested isolates.
