# Supplementary material for: Antimicrobial Resistance Profile of Klebsiella pneumoniae Clinical Isolates at a Tertiary Pulmonology-Critical Care Centre in Romania: A Five-Year Retrospective Study (2021–2026)
Source: Antibiotics (Basel). 2026 Aug 12;15(8):774. doi: 10.3390/antibiotics15080774 (PMC13509181; doi:10.3390/antibiotics15080774)
Supplement: Supplementary file 1 [file antibiotics-15-00774-s001.zip › antibiotics-4458778-supplementary.pdf]

Supplementary Materials

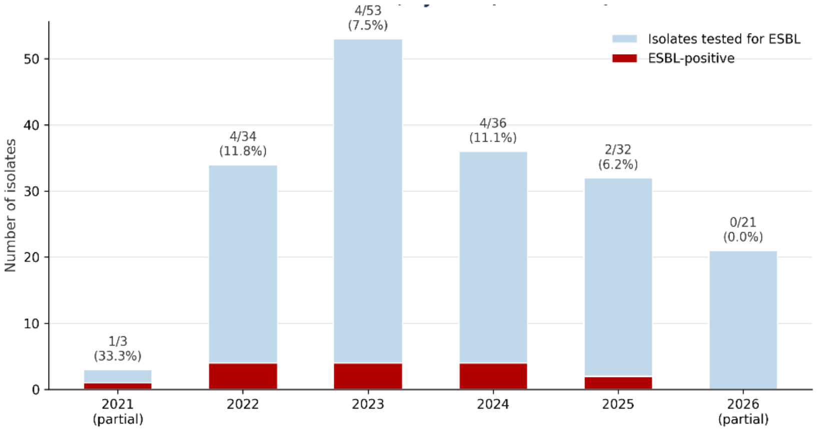

Figure S1. ESBL positivity among Klebsiella pneumoniae isolates tested for ESBL production, by year of isolation (2021–2026). Bars show the number of isolates tested per year (light blue) and the ESBL-positive subset (dark red), with the positive fraction and percentage labeled above each year. Data collection began in July 2021 and ended in May 2026; the 2021 and 2026 columns therefore reflect partial-year data.

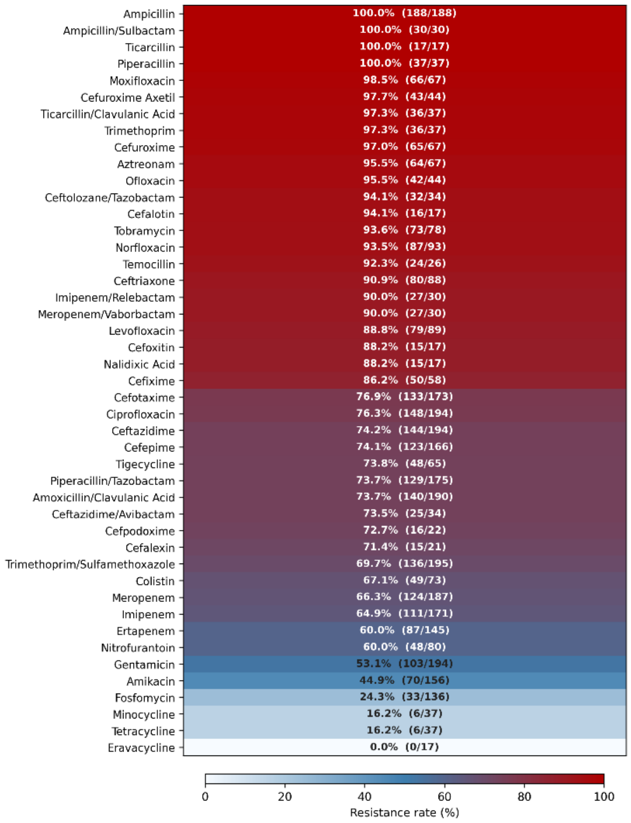

Figure S2. Resistance rate by individual antimicrobial agent among Klebsiella pneumoniae isolates (n ≥ 15 tested per agent). Cells show the percentage of isolates classified as resistant among those tested, with the corresponding fraction (resistant/tested) in parentheses; color intensity scales with resistance rate (0–100%).

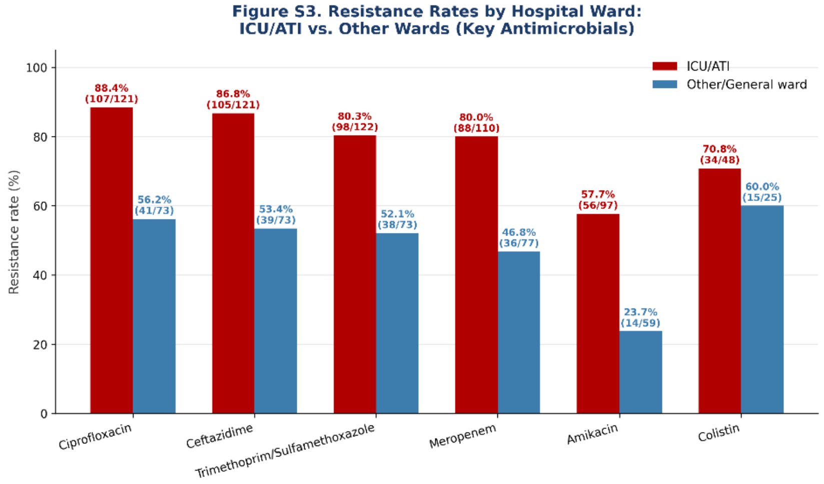

Figure S3. Resistance rates for six key antimicrobial agents, stratified by hospital ward (ICU/ATI vs. other wards). Bars show the percentage of isolates resistant among those tested, with the resistant/tested fraction labeled above each bar.

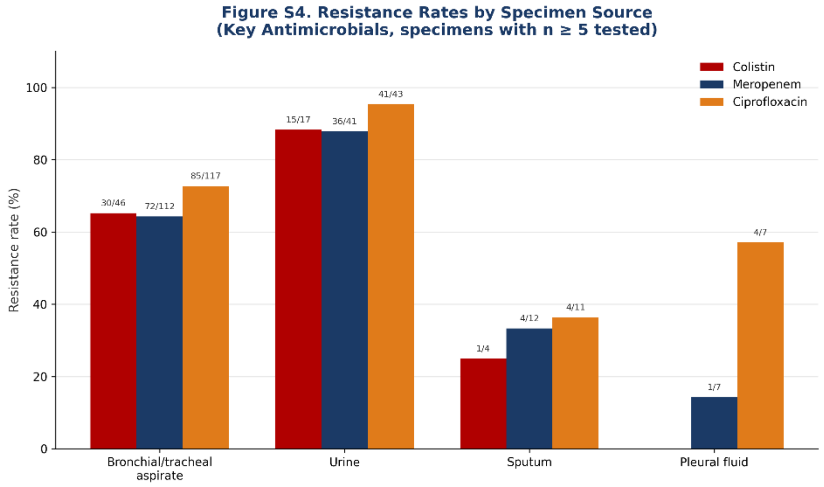

Figure S4. Resistance rates for colistin, meropenem, and ciprofloxacin, stratified by specimen source, restricted to specimen types with at least five isolates tested per agent. Fractions above each bar indicate resistant/tested isolates.
